# Ethics guidelines use and Indigenous governance and participation in Aboriginal and Torres Strait Islander health research: a national survey

**DOI:** 10.5694/mja2.51757

**Published:** 2022-10-17

**Authors:** Luke J Burchill, Aneta Kotevski, Daniel LM Duke, Jeanette E Ward, Megan Prictor, Karen E Lamb, Michelle Kennedy

**Affiliations:** ^1^ Royal Melbourne Hospital Melbourne VIC; ^2^ The University of Melbourne Melbourne VIC; ^3^ Nulungu Research Institute, the University of Notre Dame Australia Broome WA; ^4^ Centre for Epidemiology and Biostatistics the University of Melbourne Melbourne VIC; ^5^ The University of Newcastle Newcastle NSW

**Keywords:** Indigenous health, Ethics, research, Surveys and questionnaires

## Abstract

**Objectives:**

To assess the use of NHMRC Indigenous research guidelines by Australian researchers and the degree of Aboriginal and Torres Strait Islander governance and participation in Indigenous health research.

**Design, setting, participants:**

Cross‐sectional survey of people engaged in Indigenous health research in Australia, comprising respondents to an open invitation (social media posts in general and Indigenous health research networks) and authors of primary Indigenous health research publications (2015–2019) directly invited by email.

**Main outcome measures:**

Reported use of NHMRC guidelines for Indigenous research; reported Indigenous governance and participation in Indigenous health research.

**Results:**

Of 329 people who commenced the survey, 247 people (75%) provided responses to all questions, including 61 Indigenous researchers (25%) and 195 women (79%). The NHMRC guidelines were used “all the time” by 206 respondents (83%). Most respondents (205 of 247, 83%) reported that their research teams included Indigenous people, 139 reported dedicated Indigenous advisory boards (56%), 91 reported designated seats for Indigenous representatives on ethics committees (37%), and 43 reported Indigenous health research ethics committees (17%); each proportion was larger for respondents working in Indigenous community‐controlled organisations than for those working elsewhere. More than half the respondents reported meaningful Indigenous participation during five of six research phases; the exception was data analysis (reported as apparent “none” or “some of the time” by 143 participants, 58%).

**Conclusions:**

Indigenous health research in Australia is largely informed by non‐Indigenous world views, led by non‐Indigenous people, and undertaken in non‐Indigenous organisations. Re‐orientation and investment are needed to give control of the framing, design, and conduct of Indigenous health research to Indigenous people.

**The known:** The NHMRC guidelines for *Ethical conduct in research with Aboriginal and Torres Strait Islander peoples and communities* recommend advancing Indigenous oversight of and participation in Indigenous health research.

**The new:** Despite reported widespread use of the NHMRC guidelines, our survey indicated that Indigenous people are often not involved in the oversight and conduct of Indigenous health research. Their participation was more frequently reported by researchers in Indigenous community‐controlled organisations than by people in other organisations.

**The implications:** Research organisations should identify and overcome barriers that limit the oversight of and participation of Indigenous people in Indigenous health research. Investing in Indigenous community‐controlled organisations will be part of the solution.

The National Health and Medical Research Council (NHMRC) is the primary source of funding for Aboriginal and Torres Strait Islander (Indigenous) health research in Australia; it has awarded more than $320 million to Indigenous health research projects since 2015.^1^ The NHMRC guidelines for *Ethical conduct in research with Aboriginal and Torres Strait Islander peoples and communities*, published in 2018,^2^ recommend a range of actions for advancing Indigenous governance and participation in research, including empowering Indigenous leadership, employing Indigenous staff, nurturing community partnerships, and establishing Indigenous health research ethics committees. The guidelines, however, are not mandatory, and the extent to which Australian research organisations have implemented them varies.

The NHMRC *Strategic framework for improving Aboriginal and Torres Strait Islander health through research*, also published in 2018,^3^ expresses a commitment to building Indigenous participation in health research. However, the types and appropriateness of organisational support for achieving this aim are unclear. As attention to differences between principles and research practice increases, understanding how these two documents are being applied is important. We therefore undertook a national cross‐sectional survey of people engaged in Indigenous health research in Australia.

## Methods

Our major aim was to assess the use by Australian researchers of the NHMRC ethics guidelines for Indigenous health and medical research, and the extent to which Indigenous governance and participation has been achieved. We defined Indigenous governance as recognised Indigenous leadership at the executive level and an active Indigenous advisory board, an Indigenous health research ethics committee, and designated Indigenous seats on the ethics committee. Indigenous participation was assessed during six stages of research: developing the research question, designing the research plan, data collection, data analysis, dissemination of results, and reporting and publishing research findings.

### Survey development

Indigenous and non‐Indigenous members of the research team with expertise in health research, research ethics, and governance co‐designed the survey according to the principles of critical allyship^4^, ^5^ and Indigenous standpoint theory.^6^ Research questions were developed in response to gaps in knowledge about the translation of ethics guidelines into organisational action and commitment to empowering Indigenous oversight and participation in Indigenous health research, identified by Indigenous members of the research team (LB, MK) living and working at the cultural interface of Western and Indigenous health and medical research.^7^ Questions raised in Indigenous critiques of the NHMRC guidelines were considered,^8^, ^9^ as were key publications on tensions between Indigenous and Western knowledge and value systems in Australian health and medical research.^10^, ^11^, ^12^, ^13^, ^14^

A pilot online survey was developed in REDCap^15^, ^16^ and shared via an internet link with four Indigenous and three non‐Indigenous researchers (age range, 24–55 years; three men, four women) for feedback on the clarity of and their willingness to answer each question, and the time required for completing the survey. All pilot testers completed the survey (mean completion time, 10.5 minutes). Our team re‐drafted and tested the survey over several cycles of feedback and analysis until consensus was reached on the final set of fifty questions (Supporting Information).

### Sampling

To include a broad sample of people working in Indigenous health research in Australia, we applied two recruitment strategies. General respondents (group 1) received an open invitation to complete the online survey via social media posts in general and Indigenous health research networks, including the Indigenous Researcher Network (IRNet) of the Australian Health Researcher Alliance (irnet@sahmri.com), the Indigenous Data Network (data-indigenous@unimelb.edu.au), the National Aboriginal Community Controlled Health Organisation (NACCHO; https://www.naccho.org.au), and universities and research institutes (including email invitations to members of the Pro‐Vice Chancellors Indigenous Network). In our snowball recruitment strategy, respondents were encouraged to share the general link within their personal and professional networks. Multiple responses from the same internet protocol (IP) address were not included in our analysis.

As targeted respondents (group 2), we invited people who had published primary Indigenous health research articles during 2015–2019, identified in a dataset of 1597 articles generated with the Lowitja Institute search engine for Indigenous health research.^17^ We excluded 460 commentaries, case studies, study protocols, and medical education articles, as well as 161 publications without identifiable corresponding authors. The corresponding authors for the 976 primary research articles in the final dataset were invited by email to complete the survey via a personalised link that could be used only once. Each eligible author was invited only once, and invited authors could not submit responses as part of group 1.

Before access was granted to the survey, potential respondents were asked to consent to participation. They were that informed that their participation would be anonymous, and that their data would not be linked with their organisation.

### Data collection, storage, and governance

Survey data were collected, managed, and securely stored using REDCap electronic data capture tools hosted at the University of Melbourne. The approach to Indigenous data governance was informed by CARE principles for Indigenous data governance (collective benefit, authority to control, responsibility, ethics).^18^ All responses were collected anonymously and assigned unique identifiers, ensuring that responses could not be linked to individual organisations. The survey was open to both respondent groups for eight weeks (1 March – 3 May 2021).

### Data analysis

For each survey question response, we report frequencies and proportions. Statistical significance of between‐group differences was assessed in χ^2^ tests. Data were analysed in Stata Statistical Software 16, and the analysis was overseen by Indigenous members of our research team.

### Ethics approval

The University of Melbourne Office of Research Ethics and Integrity approved the study (#2020‐14611‐13369‐4).

## Results

Of 329 people who commenced the survey, 247 (75%) provided responses to all survey questions and were included in our analysis; 169 received the survey via personalised invitation from the research team (17% response rate for group 2), 44 received it via professional or social networks, and 34 received it from friends or colleagues.

### Researchers undertaking Indigenous health research

Among the 247 respondents who provided complete responses, 186 were non‐Indigenous people (75%) and 195 women (79%); 49 described themselves as early career (20%), 54 as mid‐career (22%) and 101 as senior career researchers (41%). One hundred and twenty spent at least some of their working time on Indigenous health research (49%), 51 conducted Indigenous health research all the time (21%); 238 nominated health and wellbeing as their primary research theme (96%), and 88 (35%) reported other research themes, including governance and public policy, culture and heritage, languages, native title, and land and water (Box 1). Authorship of Indigenous health research publications (manuscripts, abstracts, posters, conference presentations) was reported by 219 respondents (89%).

Box 1 **Demographic characteristics of the 247 survey respondents who provided complete survey responses**

**Table:** 

Variable	Number
Indigenous status	
Aboriginal	49 (20%)
Torres Strait Islander	5 (2%)
Aboriginal and Torres Strait Islander	3 (1%)
Other Indigenous*	4 (2%)
Non‐Indigenous	186 (75%)
Gender	
Women	195 (79%)
Men	50 (20%)
Other	2 (1%)
Age (years)	
< 25	4 (2%)
25–45	93 (38%)
46–66	136 (55%)
≥ 67	12 (5%)
Rather not say	2 (1%)
State/territory	
New South Wales	58 (23%)
Victoria	55 (22%)
Queensland	38 (15%)
Western Australia	34 (14%)
Northern Territory	32 (13%)
South Australia	20 (8%)
Australian Capital Territory	8 (3%)
Tasmania	2 (1%)
Research career stage	
Senior (10 years or more)	101 (41%)
Mid‐career (5 years to less than 10 years)	54 (22%)
Early career (less than 5 years)	49 (20%)
Graduate student (Masters, PhD)	32 (13%)
Other	9 (4%)
Indigenous community leader	1 (< 1%)
Undergraduate student	1 (< 1%)
Work time devoted to Indigenous health‐related research	
All of the time	51 (21%)
Most of the time	46 (19%)
Half of the time	30 (12%)
Some of the time	120 (49%)
Research theme^†^	
Health and wellbeing	238 (96%)
Governance and public policy	26 (11%)
Culture and heritage	28 (11%)
Languages and cultural expression	20 (8%)
Native title and traditional ownership	7 (3%)
Land and water	7 (3%)
Other	16 (6%)
Research field^†^	
Public health/health services research	195 (79%)
Clinical research	78 (32%)
Laboratory research	10 (4%)
Other	18 (7%)

* Indigenous people from other geographic regions (eg, Māori).

† Multiple responses possible.

### Organisations in which Indigenous health research is undertaken

Of the 247 respondents, 80 worked in Indigenous community‐based or ‐controlled organisations (32%) (Box 2); 185 had received cultural safety and awareness training (75%), 93 had received formal education in the history of health research involving Indigenous people (38%), 32 had received formal education in Indigenous data governance and sovereignty (13%), and 80 had received formal training in applying ethics guidelines (32%).

Box 2 Characteristics of the organisations in which the 247 respondents undertook most of their Indigenous health research*

**Table:** 

Organisation type	Number
University	129 (52%)
Indigenous community‐based organisation	80 (32%)
Research institute	62 (25%)
Hospital	34 (14%)
Non‐government organisation	19 (8%)
Government agency	17 (7%)
Mainstream primary care	16 (6%)
Advanced health and research translation centre	1 (< 1%)
Other	6 (2%)

* Participants could report more than one organisation type.

### Ethics guideline use in Indigenous health and medical research

The NHMRC guidelines were used “all the time” by 206 respondents (83%) and “some of the time” by 36 (15%); 161 respondents used additional local, state, or national guidelines (65%), including Australian Institute of Aboriginal and Torres Strait Islander Studies guidelines and code of ethics^19^ (45 respondents), the New South Wales Aboriginal Health and Medical Research Council guidelines^20^ (26 respondents), and the South Australian Aboriginal Health Research Accord^21^ (19 respondents). Use of ethics guidelines other than the NHMRC guidelines was more common among people working in community‐controlled than among those working in other organisations (60 of 80, 75% *v* 96 of 167, 57%).

### Indigenous governance and participation at the level of primary organisation

Indigenous governance was reported to be present at the highest level of their primary research organisations by 150 respondents (61%); 139 reported Indigenous advisory boards (56%), 91 reported designated ethics committee seats for Indigenous representatives (37%), and 43 reported dedicated Indigenous health research ethics committees (17%). Respondents in community‐controlled organisations more frequently reported Indigenous representation at the highest level of their organisations (58 of 80, 72%) than those in other organisations (92 of 167, 55%). Researchers working in Indigenous community‐controlled organisations also more frequently reported that Indigenous advisory boards (51 of 80, 64% *v* 88 of 167, 53%) and designated Indigenous members of research ethics committees (35 of 80, 44% *v* 56 of 167, 34%) played active roles in Indigenous oversight of health research.

Two hundred and five respondents reported that their research teams included Aboriginal or Torres Strait Islander members (83%), and 234 stated that it was very important that Indigenous researchers played a leading role in the governance of Indigenous health research projects (95%) (Box 3). Meaningful Indigenous participation during data analysis was reported to be apparent “none of the time” by 39 of 247 respondents (16%) and “some of the time” by 104 (42%); during each of the other five stages of research, it was reported as apparent by more than half the respondents (Box 4).

Box 3 **Participation of Indigenous people in Indigenous health research, and the perceived importance of such participation**

**Table:** 

Survey question	Responses
Total number of respondents	247
Do you currently have Aboriginal and/or Torres Strait Islander people on your research team?	
Yes	205 (83%)
No	20 (8%)
Not currently undertaking Indigenous health research	22 (9%)
How important is it that Aboriginal and/or Torres Strait Islander researchers play a leading role in the governance of Aboriginal and/or Torres Strait Islander research?	
Very important	234 (95%)
Somewhat important	11 (4%)
Not important	2 (1%)

Box 4 Meaningful Indigenous participation in Indigenous health research, by research phase*

**Figure:**
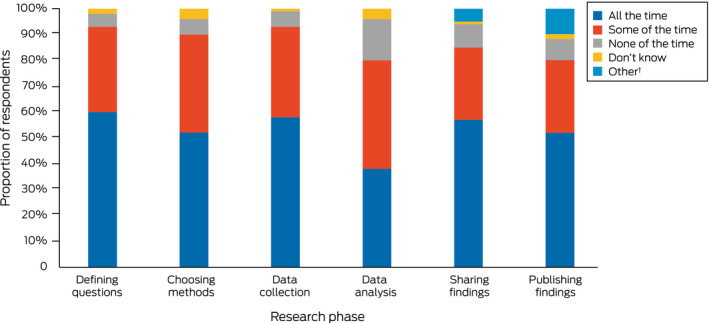
Full summary of responses is included in the table in the Supporting Information. * Total number of respondents: 247. † “I have not yet been at the reporting and publication/disseminating of results stage of an Indigenous research project.”

### Consent in Aboriginal and Torres Strait Islander research

Of the 239 people who answered survey questions about consent in Aboriginal and Torres Strait Islander health research, 119 were satisfied (50%) and 94 somewhat satisfied (39%) that the current approach to consent minimised power imbalances between researchers and participants; 18 were not satisfied (8%), eight did not know (3%). Similarly, 131 of 239 respondents were satisfied (55%) and 86 somewhat satisfied (36%) that current approaches to consent respects Indigenous groups rights and community decision‐making processes; 14 were not satisfied (6%), eight did not know (3%).

## Discussion

The fundamental question raised by our national survey is: how can Indigenous health research benefit Indigenous people without meaningful oversight and participation by Indigenous people? We found that the NHMRC guidelines for Indigenous research are widely used, but that local guidelines are also applied, reflecting local community expectations regarding Indigenous health research. However, our survey findings suggest that barriers to translating the NHMRC guidelines into research practice remain. These include inadequate levels of education about applying the guidelines, the history of Indigenous health research in Australia, and Indigenous governance and data sovereignty. Most importantly, we found that Indigenous governance and participation was inadequate at each stage of research.

Researchers in Indigenous community‐based organisations more frequently reported Indigenous oversight and use of national and state and local ethics guidelines. By providing Indigenous governance and oversight, community‐controlled organisations may not only better align their research practices with the NHMRC guidelines, but also be more willing to use local ethics guidelines that reflect community expectations about Indigenous health research.

Only 25% of our survey respondents were Indigenous people, and 32% undertook Indigenous health research in Indigenous community‐based organisations. Ninety per cent of those currently undertaking Indigenous health research (205 of 225 respondents) reported that their research teams included Aboriginal or Torres Strait Islander people, but many of those working in non‐Indigenous organisations reported that their organisations did not have structures or processes for Indigenous oversight (88 of 167, 53%) or ethics review (56 of 167, 40%). As such, the focus of investment for organisations undertaking Indigenous health research in Australia appears to be at the individual level (employing people to undertake research) rather than at the organisational level (investing in structures and processes for engaging and empowering Indigenous people to have oversight of Indigenous health research).

Indigenous participation was infrequently reported for the data analysis phase of research (“all of the time”: 38%). Information is informed by the context in which it is generated, collected, analysed, and shared. The risks of data analysis without community oversight or control have been recognised by Indigenous scholars who advocate Indigenous data sovereignty to offset views of Indigenous people that reflect what has been characterised as “5D data” (dominated by disparity, deprivation, disadvantage, dysfunction, and difference); the “primary problematic is that the Indigenous ways of seeing the world are not doing the shaping”.^22^ While Indigenous leaders have called for greater investment in Indigenous statistical capacity,^23^ only 32 people (13%) in our survey had received formal education in Indigenous data governance and sovereignty. This suggests that a significant proportion of data analysis is undertaken by people and research teams not familiar with Indigenous governance and data sovereignty principles, leading to publications that reflect Western scientific paradigms at the expense of Aboriginal and Torres Strait Islander world views.^24^

Increasing Indigenous governance and participation will require fundamental change to how Indigenous health research is conducted. We join with generations of Aboriginal and Torres Strait Islander researchers, community leaders, organisations and allies who continue to fight for change in how Indigenous health research is governed. Together, we recognise that, despite calls for self‐determination by Aboriginal and Torres Strait Islander people and in ethics guidelines, the level of their participation in and oversight of Indigenous health research in Australia fall short of what is recommended by national guidelines.

### Limitations

As we could not accurately characterise the entire sample who received the online survey, and surveys are more likely to be completed by people interested in the subject, the representativeness of our survey sample is unknown. However, as half the respondents devoted at least half their time to Indigenous health‐related research, and most had published in the field, our findings reflect the perspectives of a large sample of researchers in Indigenous health research. More than three‐quarters of respondents were women, but we cannot conclude that the Indigenous health workforce is mostly female, although such a conclusion would be consistent with our experience.

### Conclusion

Despite widespread use of the NHMRC guidelines, Indigenous health research in Australia is largely informed by non‐Indigenous world views, led by non‐Indigenous people, and undertaken in non‐Indigenous organisations. Organisations based in and controlled by Aboriginal and Torres Strait Islander communities are more likely to have research practices that are aligned with the NHMRC guidelines and reflect community expectations regarding Indigenous governance and participation in research. Re‐orientation and investment are needed to give control of the framing, design, and conduct of Indigenous health research to Indigenous people.

## Open access

Open access publishing facilitated by The University of Melbourne, as part of the Wiley – The University of Melbourne agreement via the Council of Australian University Librarians.

## Competing interests

No relevant disclosures.

## Supporting information




**Table S1** Meaningful Indigenous participation in Indigenous health research, by research phase, as reflected in survey responses by 247 participants (survey questions 3.13 to 3.18)

Appendix S1
