# Supplementary material for: Ethics guidelines use and Indigenous governance and participation in Aboriginal and Torres Strait Islander health research: a national survey
Source: Med J Aust. 2022 Oct 17;218(2):89–93. doi: 10.5694/mja2.51757 (PMC10952733; doi:10.5694/mja2.51757)
Supplement: Supplementary file 2 — Appendix S1 [file MJA2-218-89-s002.pdf]

National Survey of Australian Health Researchers Exploring Guidelines, Governance and Consent for Research involving Aboriginal and Torres Strait Islander communities

THANK YOU for taking the time to complete this survey. It is open to BOTH Indigenous and non-Indigenous Australian researchers undertaking Aboriginal and Torres Strait Islander health and medical research. All responses will be kept private and confidential.

Background

Prior research has focused on the content and meaning of the National Health and Medical Research (NHMRC) Guidelines. In contrast, this Indigenous-led survey seeks to understand how the guidelines are being applied by Indigenous and non-Indigenous people engaged in Indigenous health research in Australia. Additionally, it will also explore the organisational context and governance arrangements in place for Indigenous health research.

This survey will take approximately 10-15 minutes to complete.

By proceeding with this survey, you are providing consent to participate.<br>Do you consent?	◯ Yes<br>◯ No

Section 1. Researcher and Organisation Profiles
Your answers help us understand the profile of researchers undertaking Aboriginal and Torres Strait Islander research in Australia.

1.1 How did you hear about this survey?

- ◯ Through my professional or social network listings
- ◯ Personal email invitation from research team
- ◯ Shared by a friend or colleague

1.2 Which of the following themes does your Aboriginal and Torres Strait Islander research explore? (Choose all that apply)

- ☐ Culture and heritage
- ☐ Governance and Public policy
- ☐ Health and wellbeing
- ☐ Land and water
- ☐ Languages and cultural expression
- ☐ Native Title and traditional ownership
- ☐ Other

(Themes as outlined by the Australian Institute of Aboriginal and Torres Strait Islander Studies)

1.2.1 Research theme other

____

1.3 Which academic focus best describes your field of research? (Choose all that apply)

- ☐ Lab based experimental research
- ☐ Clinical research including case series, cohort studies, clinical trials and outcomes
- ☐ Public health and health services research
- ☐ Other

1.3.1 Research field other

____

1.4 In what state or territory of Australia is your primary research organisation based?

- ◯ NSW
- ◯ VIC
- ◯ TAS
- ◯ QLD
- ◯ NT
- ◯ WA
- ◯ SA
- ◯ ACT

1.5 Which age range applies to you?

- ◯ < 25 years old
- ◯ 25-45 years old
- ◯ 46-66 years old
- ◯ >67 years old
- ◯ Rather not say

1.6 Which of the following best describes your gender identity?

- ◯ Male
- ◯ Female
- ◯ Gender diverse/Non-binary
- ◯ Intersex
- ◯ Other
- ◯ Rather not say

1.7 Do you identify as:

- ◯ Aboriginal
- ◯ Torres Strait Islander
- ◯ Aboriginal and Torres Strait Islander
- ◯ Indigenous (but not Aboriginal or Torres Strait Islander)
- ◯ Non-Indigenous

1.8 What best describes your career stage?

◯ Undergraduate student
◯ Graduate student (Masters or PhD)
◯ Early career within 5 years of contributing to Indigenous health research
◯ Mid-career >5 but < 10 years
◯ Senior career >10 years
◯ Indigenous Community Leader
◯ Other

1.8.1 Career stage other

____

1.9 What amount of time do you spend conducting research of any kind?

◯ Some of the time
◯ Half of the time
◯ Most of the time
◯ All of the time

1.10 What amount of time do you spend conducting Aboriginal and Torres Strait Islander research?

◯ Some of the time
◯ Half of the time
◯ Most of the time
◯ All of the time

1.11 What amount of your research time is conducted on country with Aboriginal and Torres Strait Islander communities who are the research focus?
This applies equally to urban, regional and remote communities who are the focus the research being conducted.

◯ Some of the time
◯ Half of the time
◯ Most of the time
◯ All of the time
◯ None of the time

1.12 Do you primarily undertake your Aboriginal and Torres Strait Islander research at an Indigenous organisation?

◯ Yes
◯ No
◯ Don't know

1.13 In which organisation do you undertake the majority of your Aboriginal and Torres Strait Islander research?
Choose all that apply

☐ University
☐ Hospital
☐ Research Institute
☐ Government Agency
☐ Aboriginal community-based organisation (i.e. health service, land council)
☐ Mainstream primary care setting
☐ Non-government organisation (NGO)
☐ Advanced health and research translation centre
☐ Other (please specify)

1.13.0 Organisation other - please specify

____

1.14 Have you previously been listed as an author on research published relating to Aboriginal or Torres Strait Islander health in Australia.
This may include manuscripts, abstracts, posters and conference presentations

◯ Yes
◯ No

Section 2. Ethical Guidelines for Aboriginal and Torres Strait Islander Research.
We would like to understand how the NHMRC guidelines are applied by those undertaking Aboriginal and Torres Strait Islander research.

2.1 Do you use the NHMRC's Ethical conduct in research with Aboriginal and Torres Strait Islander Peoples and communities: Guidelines for researchers and stakeholders when undertaking research with Aboriginal and Torres Strait Islander people?

- ◯ All of the time
- ◯ Some of the time
- ◯ None of the time

2.2 Aside from the NHMRC Guidelines, do you use any other ethical guidelines when undertaking Aboriginal and Torres Strait Islander research?

- ◯ Yes - please specify
- ◯ No

2.2.1 Other guidelines

____

2.3 Does your primary organisation's ethics committee refer to the NHMRC Guidelines when reviewing Aboriginal and Torres Strait Islander ethics applications?

- ◯ Yes
- ◯ No
- ◯ My primary organisation doesn't have an ethics committee
- ◯ I don't know

2.4 Thinking about your most recent research project involving Aboriginal and Torres Strait Islander people, at what stage/s of research did you refer to the NHMRC's Ethical conduct in research with Aboriginal and Torres Strait Islander Peoples and communities: Guidelines for researchers and stakeholders? Choose all that apply

- ☐ Building relationships with community
- ☐ Developing the research idea
- ☐ Developing the research project methods
- ☐ Designing governance mechanisms and seeking agreement
- ☐ Data collection
- ☐ Analyzing the data and making sense of the findings
- ☐ Report writing
- ☐ Vetoing publications
- ☐ Sharing and translating the results in action

2.5 At what stage/s of research do you consider the NHMRC's Ethical conduct in research with Aboriginal and Torres Strait Islander Peoples and communities: Guidelines for researchers and stakeholders to be MOST IMPORTANT?
Choose all that apply

- ☐ Building relationships with community
- ☐ Developing the research idea
- ☐ Developing the project and seeking agreement
- ☐ Data collection
- ☐ Analyzing the data and making sense of the findings
- ☐ Report writing
- ☐ Sharing and translating the results in action
- ☐ I don't use NHMRC guidelines

2.6 Prior to commencing any research, do you formally meet and discuss NHMRC Guidelines with the Indigenous community that is the focus of your research?

- ◯ Yes
- ◯ No
- ◯ Sometimes
- ◯ Not sure
- ◯ I don't use NHMRC guidelines

2.7 How important are NHMRC's Ethical conduct in research with Aboriginal and Torres Strait Islander Peoples and communities: Guidelines for researchers and stakeholders and Keeping research on track II for your Aboriginal and Torres Strait Islander health research practice?

- ◯ Very important
- ◯ Sometimes important
- ◯ Not important
- ◯ Not sure
- ◯ I don't use NHMRC guidelines

2.8 Have you received any formal training in how to apply NHMRC's Ethical conduct in research with Aboriginal and Torres Strait Islander Peoples and communities: Guidelines for researchers and stakeholders or Keeping research on track II to your research practice?

- ◯ Yes - please provide name of training program
- ◯ No

2.8.1 Training_Organisation_Other

____

2.9 How satisfied are you that NHMRC's Ethical conduct in research with Aboriginal and Torres Strait Islander Peoples and communities: Guidelines for researchers and stakeholders safeguard Aboriginal and Torres Strait Islander people from the risks of research?

- ◯ Satisfied
- ◯ Not satisfied
- ◯ Not sure
- ◯ I don't use the NHMRC guidelines

2.10 In your opinion, who is MOST responsible for ensuring Aboriginal and Torres Strait Islander research is conducted ethically?
Choose all that apply

- ☐ Governments and their agencies (i.e. NHMRC) funding Aboriginal and Torres Strait Islander Research
- ☐ Organisations that undertake Aboriginal and Torres Strait Islander Research
- ☐ Human research ethics committees approving Aboriginal and Torres Strait Islander Research
- ☐ Researchers undertaking Aboriginal and Torres Strait Islander Research
- ☐ Aboriginal and Torres Strait Islander researchers undertaking Indigenous research
- ☐ Aboriginal and Torres Strait Islander people leading Aboriginal and Torres Strait Islander research
- ☐ Aboriginal and Torres Strait Islander research participants
- ☐ Aboriginal Community Controlled Health Organisations
- ☐ Other

2.10.1 Responsible for ethics - other

____

Section 3. Governance
We would like to understand governance arrangements in your primary organisation* and how this influences your ability to effectively undertake Aboriginal and Torres Strait Islander research. Governance is the way your organisation is managed, directed and held accountable for achieving its goals. Governance arrangements include the structures, systems and process that control how your organisation operates. This can include your board, constitution, sub-committees and policies.

***Primary organisation refers to the main organisation you conduct your Aboriginal and Torres Strait Islander research in.**

3.1 Does your primary organisation have Aboriginal and Torres Strait Islander representatives at its highest level (Executive/Board/Senate)?

- ◯ Yes
- ◯ No
- ◯ Don't know

3.2 Does your primary organisation have an Aboriginal and/or Torres Strait Islander advisory board or advisory council that is involved in your Aboriginal and Torres Strait Islander research?

- ◯ Yes
- ◯ No
- ◯ Don't know

3.3 Does your primary organisation have delegated seats for Aboriginal and Torres Strait Islander people on its ethics committee?

- ◯ Yes
- ◯ No
- ◯ Don't know
- ◯ My primary organisation doesn't have an ethics committee

3.4 Does your primary organisation have its own Aboriginal and/or Torres Strait Islander research ethics committee?

- ◯ Yes
- ◯ No
- ◯ Don't know

3.5 Does your primary organisation have its own policy or framework to guide researchers undertaking Aboriginal and/or Torres Strait Islander research?

- ◯ Yes
- ◯ No
- ◯ Don't know

3.6 Do you currently have Aboriginal and/or Torres Strait Islander people on your research team?

- ◯ Yes
- ◯ No
- ◯ Don't know
- ◯ I am not currently undertaking an Indigenous project

3.7 Has your primary organisation provided you with any formal education in the history of health and medical research with Aboriginal and Torres Strait Islander people?

- ◯ Yes
- ◯ No
- ◯ Don't know

3.8 Has your primary organisation provided you with any formal cultural safety and awareness training?

- ◯ Yes
- ◯ No
- ◯ Don't know

3.9 Does your primary organisation provide formal education in the National Statement on Ethical Conduct in Human Research (2007) and other applicable guidelines/statements to your research?

- ◯ Yes
- ◯ No
- ◯ Don't know

3.10 Has your primary organisation provided you with any formal education or training in Indigenous data governance and/or sovereignty?

- ◯ Yes
- ◯ No
- ◯ Don't know

3.11 In your opinion, how important is it that Aboriginal and/or Torres Strait Islander researchers play a leading role in the governance of Aboriginal and/or Torres Strait Islander research?

- ◯ Very important
- ◯ Somewhat important
- ◯ Not important
- ◯ Don't know

3.12 Thinking about your Indigenous research project/s, to what extent has there been meaningful participation by Aboriginal and/or Torres Strait islander people in establishing governance arrangements?

- ◯ All of the time
- ◯ Some of the time
- ◯ None of the time
- ◯ I don't know

3.13 Thinking about your Indigenous research project/s, to what extent has there been meaningful participation by Aboriginal and/or Torres Strait islander people in developing the research idea?

- ◯ All of the time
- ◯ Some of the time
- ◯ None of the time
- ◯ I don't know

3.14 Thinking about your Indigenous research project/s, to what extent has there been meaningful participation by Aboriginal and/or Torres Strait islander people in designing research methods?

- ◯ All of the time
- ◯ Some of the time
- ◯ None of the time
- ◯ I don't know

3.15 Thinking about your Indigenous research project/s, to what extent has there been meaningful participation by Aboriginal and/or Torres Strait islander people in data collection?

- ◯ All the time
- ◯ Some of the time
- ◯ None of the time
- ◯ I don't know

3.16 Thinking about your Indigenous research project/s, to what extent has there been meaningful participation by Aboriginal and/or Torres Strait islander people in data analysis?

- ◯ All of the time
- ◯ Some of the time
- ◯ None of the time
- ◯ I don't know

3.17 Thinking about your Indigenous research project/s, to what extent has there been meaningful participation by Aboriginal and/or Torres Strait islander people in reporting and publication?

- ◯ All of the time
- ◯ Some of the time
- ◯ None of the time
- ◯ I have not yet been at the reporting and publication stage of an Indigenous research project
- ◯ I don't know

3.18 Thinking about your Indigenous research project/s, to what extent has there been meaningful participation by Aboriginal and/or Torres Strait islander people in disseminating the results of your research?

- ◯ A lot of the time
- ◯ Some of the time
- ◯ None of the time
- ◯ I have not yet been at the disseminating of results stage in an Indigenous research project
- ◯ I don't know

Section 4. Consent in Aboriginal and Torres Strait Islander Research
The NHMRC's Ethical conduct in research with Aboriginal And Torres Strait Islander Peoples and communities: Guidelines for researchers and stakeholders, stipulates that all people have the right to free, prior and informed consent in all aspects of the research process and that no one party has more power or strength than any other; that Indigenous groups or community decision-making processes should be allowed to operate, and that Indigenous peoples' right to choose how they want to live is respected.

4.1 How satisfied are you that your current approach to consent minimises the likelihood of power imbalances between researcher and participant?

- ◯ Satisfied
- ◯ Somewhat satisfied
- ◯ Not satisfied
- ◯ Don't know

4.2 How satisfied are you that your current approach to consent respects Indigenous groups rights and community decision-making processes?

- ◯ Satisfied
- ◯ Somewhat satisfied
- ◯ Not satisfied
- ◯ Don't know

4.3 How satisfied are you that your current approach to consent respects Indigenous peoples' right to choose how they want to interact with researchers?

- ◯ Satisfied
- ◯ Somewhat satisfied
- ◯ Not satisfied
- ◯ Don't know

4.5 Have you found that arriving at appropriate consent arrangements for your projects involving Aboriginal and Torres Strait Islander people is more complicated than in your other research projects?

- ◯ Yes
- ◯ No
- ◯ Sometimes

4.4 When deciding on consent processes for your research projects, has there been meaningful participation of Aboriginal and/or Torres Strait Islander people in the design of those processes (the manner in which consent is explained to and given by Aboriginal and Torres Strait Islander people)?

- ◯ Yes
- ◯ No
- ◯ Sometimes

4.6 To what extent have you modified standard consent arrangements in your Aboriginal and Torres Strait Islander research?

- ◯ Significantly modified
- ◯ Somewhat modified
- ◯ Almost no modification
- ◯ No modification

4.7 If you have modified consent arrangements for your Aboriginal and Torres Strait Islander research, how often have you done this?

- ◯ All research projects
- ◯ Some research projects
- ◯ I have never modified consent arrangements

Thank you for your time responding to this survey. Is there anything else you might like to tell us?

____
